# QM Cluster or QM/MM in Computational Enzymology: The Test Case of LigW-Decarboxylase

**DOI:** 10.3389/fchem.2018.00249

**Published:** 2018-06-28

**Authors:** Mario Prejanò, Tiziana Marino, Nino Russo

**Affiliations:** Dipartimento Di Chimica e Tecnologie Chimiche, Università della Calabria, Rende, Italy

**Keywords:** QM, QM/MM, decarboxylation, enzymatic catalysis, reaction mechanism, LigW

## Abstract

The catalytic mechanism of the decarboxylation of 5-carboxyvanillate by LigW producing vanillic acid has been studied by using QM cluster and hybrid QM/MM methodologies. In the QM cluster model, the environment of a small QM model is treated with a bulky potential while two QM/MM models studies include partial and full protein with and without explicitly treated water solvent. The studied reaction involves two sequential steps: the protonation of the carbon of the 5-carboxy-vanillate substrate and the decarboxylation of the intermediate from which results deprotonated vanillic acid as product. The structures and energetics obtained by using three structural models and two density functionals are quite consistent to each other. This indicates that the small QM cluster model of the presently considered enzymatic reaction is appropriate enough and the reaction is mainly influenced by the active site.

## Introduction

Enzymes are biological machines that efficiently catalyze a huge number of chemical reactions in the very short time steps required by the physiological processes. In the last decades, computational enzymology has become a very useful tool for studying enzyme activity since it allows to determine the energies and structures of short-lived intermediates and transition states. Through computational enzymology, different reaction pathways can be analyzed, and their feasibility can be established by a careful analysis of calculated energy barriers. A crucial issue in computational enzymology is the choice of the model to be used in the simulations. The choice is not so obvious because it depends on the nature of enzyme (without or with metal cofactor) on the catalytic pocket and on the amino acids implicated in the chemical reaction. In fully quantum mechanical (QM) treatment (Himo, 2006; Ramos and Fernandes, 2008; Siegbahn and Himo, 2011; Merz, 2014), a cluster that contains all the residues around the active site is considered.

In metalloenzymes, the construction of the cluster model is facilitated by the presence of metal ions and all the residues of their inner coordination sphere. One may also need to include some other surrounding residues involved in the chemical process. Atoms at the periphery of the model, where truncation is made, are normally frozen in their original positions present in the crystallographic structure for avoiding artificial expansion or other rearrangements (Blomberg et al., 2014). The surrounding protein environment not directly implicated in the chemical transformation, is modeled with implicit dielectric constant-based solvation models (Warshel, 1991). This method is highly versatile and widely applied to a large variety of enzyme families and to different classes of enzymes (Ramos and Fernandes, 2008; Liao et al., 2010; Amata et al., 2011a; Himo, 2017; Piazzetta et al., 2017; Prejanò et al., 2017a). A different approach developed in 1976 (Warshel and Levitt, 1976) is the hybrid quantum mechanics/molecular mechanics (QM/MM) (Senn and Thiel, 2009; Quesne et al., 2016; Ryde, 2016). In this procedure, other than the QM portion a large number of residues (or the whole enzyme sequence) is treated at molecular mechanics level (MM) (Senn and Thiel, 2009). Convergence studies performed by different research groups indicated that QM-cluster models (Siegbahn and Himo, 2011; Ryde, 2017) gives reliable energetics when the size of the model is large enough. Herein we perform a theoretical study using both QM cluster and QM/MM approaches on the gene product of LigW of 5-carboxyvanillate decarboxylase (5CVA) (Vladimirova et al., 2016). The QM part in all the models has been treated in the framework of density functional theory (DFT) and by using two different exchange-correlation functionals.

The LigW belongs to the amidohydrolase (AHS) superfamily including a high number of enzymes catalyzing the hydrolysis of a wide range of substrates. In all AHS members, a mononuclear or binuclear metal binding site is found (Gerlt and Babbitt, 2001; Seibert and Raushel, 2005). All AHS members have a (β/α)_8_-barrel structural fold and catalyze the metal-dependent hydrolysis of phosphate and carboxylate esters (Jackson et al., 2005; Shapir et al., 2006; Elias et al., 2008; Khurana et al., 2009; Duarte et al., 2011; Tobimatsu et al., 2013). LigW catalyzes the C-C bond cleavage of 5-CV to vanillate (VAN) in an oxidant-independent fashion. The 5-carboxyvanillate (5-CV) represents one of the final product of the multienzymatic degradation of the biphenyl lignin derivatives. The lignin degradation of microbial origin represents an interesting process from both commercial and biotechnological point of view owing to the plant biomass conversion in renewable aromatic chemicals and biofuels (Liu and Zhang, 2006). Furthermore, decarboxylation represents a process of widespread occurrence in nature and therefore it is of relevant biological interest (Faponle et al., 2016).

## Computational methods

All the calculations were carried out by using the Gaussian 09 program (Gaussian 09, Revision D.01, 2011)^1^. The QM portions were treated with the B3LYP (Lee et al., 1988; Becke, 1993) hybrid density functional. 6-31+G(d,p) basis set was used for the C, N, O, and H atoms, whereas the SDD pseudo-potential and corresponding orbital basis set (Andrae et al., 1990) were employed for Mn atom. Our own N-layered integrated molecular orbital and molecular mechanics (ONIOM) method was applied as the QM/MM method in the framework of electronic embedding scheme, in which the effects of the fixed MM charges are incorporated in the QM hamiltonian (Svensson et al., 1996; Vreven et al., 2006). As shown in Figure S1, the enzyme-substrate complex (ES) is a high-spin sextet species while its low-spin doublet and the intermediate-spin quartet states are energetically not accessible. The sextet state does not suffer from any spin contamination (<S^2^> equal to 8.75). The optimized minima and transition states on the potential energy surfaces were confirmed by the analysis of the corresponding Hessian matrices. Zero-point-energy corrections were calculated and added to the final energies. In order to obtain more accurate energies, single point calculations on the optimized structures were performed with the larger basis set 6-311+G(2d,2p) taking into account the effects of the protein environment by using the solvation model density (SMD) (Marenich et al., 2009), with a dielectric constant (ε = 4) of the enzyme environment, for the cluster simulations (Alberto et al., 2010; Liao et al., 2010; Amata et al., 2011a,b; Himo, 2017; Piazzetta et al., 2017; Prejanò et al., 2017a,b). Energetics presented includes D3 dispersion correction (Grimme et al., 2011). To evaluate the effect of the exchange-correlation functionals single point calculations on the B3LYP optimized geometries have been performed by using the M06-L functional that was previously demonstrated to be accurate for describing metal containing systems properties (Zhao and Truhlar, 2006, 2008) (see Table S1). NBO analysis (NBO, version 3.1, 2001)^2^ was performed on all intercepted stationary points at QM and QM/MM levels with B3LYP functional. Furthermore, the noncovalent interactions on the minima of the PES have been assessed by using the NCIPLOT tool (NCIPLOT, version 3.0, 2011)^3^.

## Computational setup and QM model definitions

**Figure 1:**
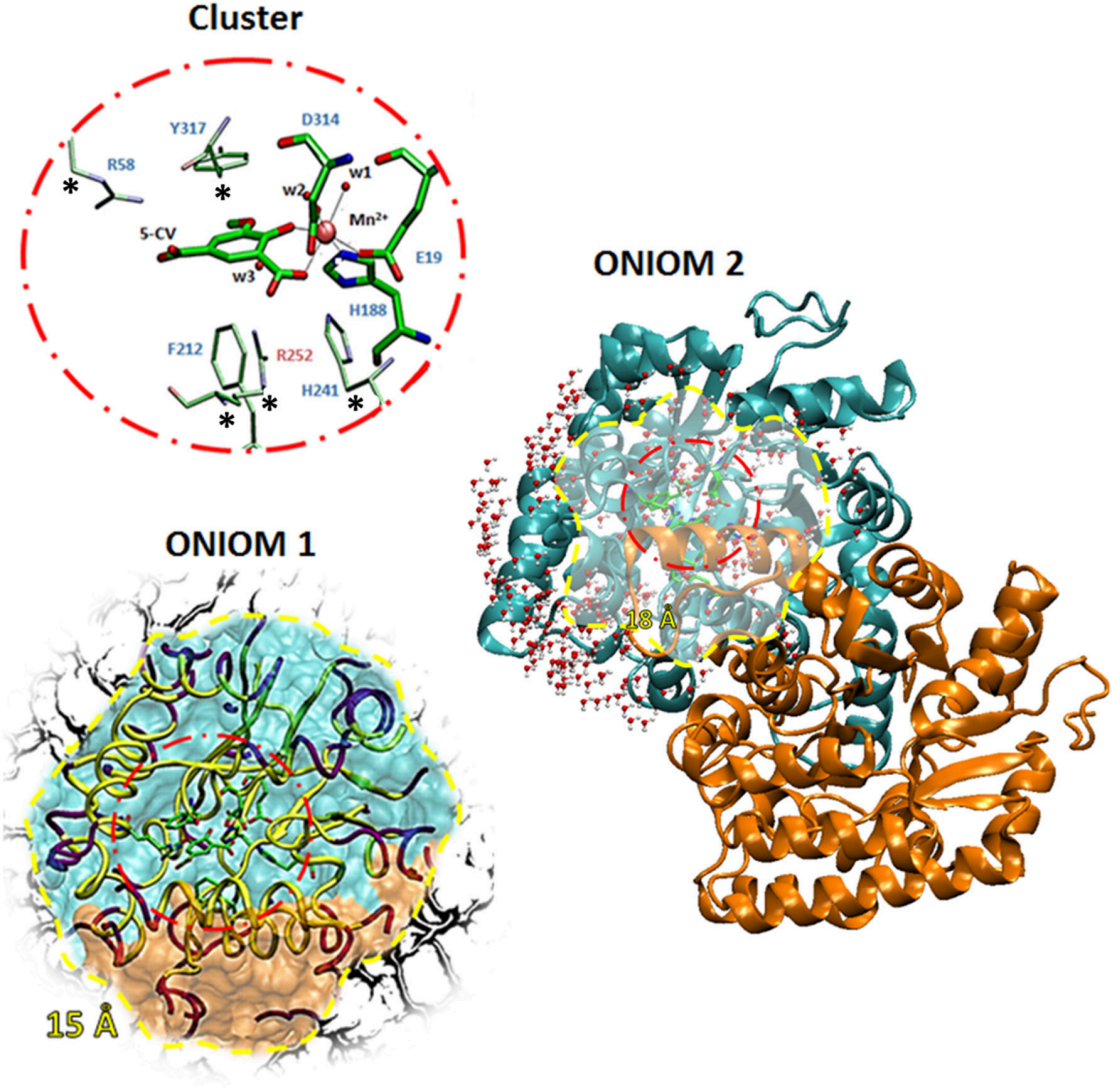
The three models used. The QM portion used in both cluster and QM/MM calculations are shown inside a red circle while the optimized region of QM/MM calculations are in yellow circle. The inner coordination shell of Mn^2+^ is shown with larger sticks on the QM portion.

The model of the LigW active site, used for both QM and QM/MM calculations, was obtained from the three-dimensional structure of wild-type LigW in the presence of the substrate-like inhibitor 5-nitrovanillate (5-NV) isolated by *N. aromaticivorans* (PDB id: 4QRN, resolution: 1.07 Å). Vladimirova et al. (2016) due to the very small difference (one atom) between the inhibitor (5-NV) and substrate (5-CV). This choice has been already shown sufficient when structurally compared with larger QM clusters (Sheng et al., 2017). In the active site, (see Figure 1) the manganese ion is octahedrally coordinated to Glu-19, His-188, Asp-314, one water molecule **w1** and the substrate. Two water molecules, (**w2** and **w3**), located at about 5 A from the substrate and other residues of the active site pocket not directly bound to the metal ion are retained in QM region (Arg58, Phe212, His241, Arg252, and Tyr317).

In the QM/MM models, the Amber ff14SB force field (Maier et al., 2015) as implemented in AMBER16 software was used. The missing MM parameters for the substrate 5-CV were created from single molecule optimization at HF/6-31G(d) level of theory with the Antechamber tool, as implemented in AMBER16 (AMBER version 16, 2016)^4^. At this purpose the General Amber Force Field (GAFF) (Wang et al., 2004) and the Restrained Electrostatic Potential (RESP) (Bayly et al., 1993) methods were used to derive intramolecular and Lennard-Jones parameters and atomic charges, respectively (see Table S2).

### QM cluster

All the amino acids of the QM region were truncated at the α-carbons, and hydrogen atoms were added manually. In order to avoid unrealistic movements of the groups during the geometry optimizations, the truncated α-carbons of the outer coordination shell labeled by stars in Figure 1 were kept fixed to their crystallographic positions. The residues were modeled according to standard procedure (Liao et al., 2010; Amata et al., 2011a,b; Siegbahn and Himo, 2011; Blomberg et al., 2014; Himo, 2017; Prejanò et al., 2017a) considering the protonation states coming from the experimental evidences (Vladimirova et al., 2016). The obtained model consists of 126 atoms with a total charge equal to zero. The size of the cluster is adequate enough to represent the chemistry involved in the considered reaction mechanisms for formation or breaking bonds.

### ONIOM-1

In this model, the QM region is surrounded by the residues present in radius of 15 Å from the metal ion center. In this way, the interactions between α and β subunits of the homodimer were included. Inside the considered sphere, an outer shell of residues with a thickness of 2 Å was fixed, and only the inner 13 Å shell was allowed to move during the QM/MM geometry optimizations. This strategy is commonly used to avoid drifting through multiple minima unrelated to the reaction coordinate. This model includes in the MM region also a number of water molecules (20) present in the crystallographic structure. The obtained model consists of 2,154 atoms with 118 atoms in QM region (Figure 1).

### ONIOM-2

A rectangular box was used to solvate the system up to 12.0 Å of the metal center. During the optimizations, all the water molecules and protein atoms in the 18 Å from the active site were kept frozen, as proposed by a recent work (Medina et al., 2017). The final model contains 11,895 atoms with 118 atoms of QM region. In this case, the MM region includes the whole protein and a number of water molecules within 5 Å around of catalytic domain as depicted in Figure 1.

## Results and discussion

**Figure 2:**
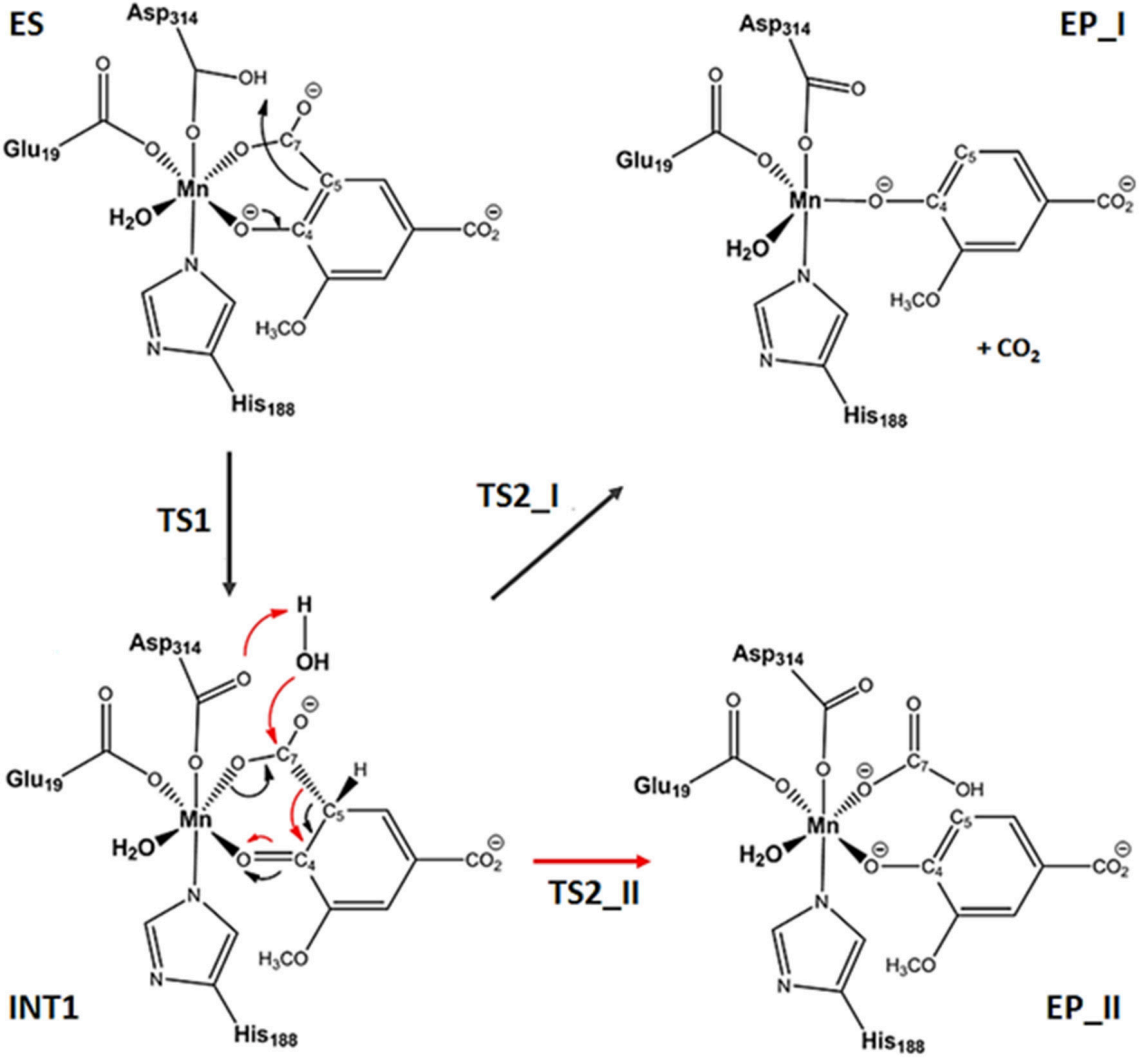
Reaction mechanism of the catalyzed by LigW. For the decarboxylation step, CO_2_ and ${\text{HCO}}_{3}^{-}$ formations occur through TS2_I and TS2_II transition states.

The reaction can follow two paths with the formation of **CO**_2_ or **HCO**_3_^−^ products (see Figure 2). After the formation of the **ES**, the reaction proceeds with the proton transfer from Asp314 to C5 of the substrate generating the **INT1** species, that acts as common intermediate for the formation of **EP_I** or **EP_II** complexes in which **CO**_2_ or ${\text{HCO}}_{3}^{-}$ product should be released. In both decarboxylation pathways, it is clear that the enzyme must generate an adjacent electron sink (such as the ketone carbonyl C4 since the formation of the new carbon–hydrogen bond) to stabilize the incipient carbanion at C5 prior to decarboxylation. This mechanism corresponds to that explored in the recent combined experimental and theoretical work (Sheng et al., 2017) where the membrane inlet mass spectrometry (MIMS) based assay is applied to study the LigW mechanism. The above-mentioned MIMS-based strategy (Sheng et al., 2017) was able to establish **CO**_2_ and not ${\text{HCO}}_{3}^{-}$ as reaction product. We have considered also the path for the bicarbonate release but our calculated PESs with the three models used give very high energetic barriers (see Table S1) that are not compatible with the enzymatic kinetics.

**Figure 3:**
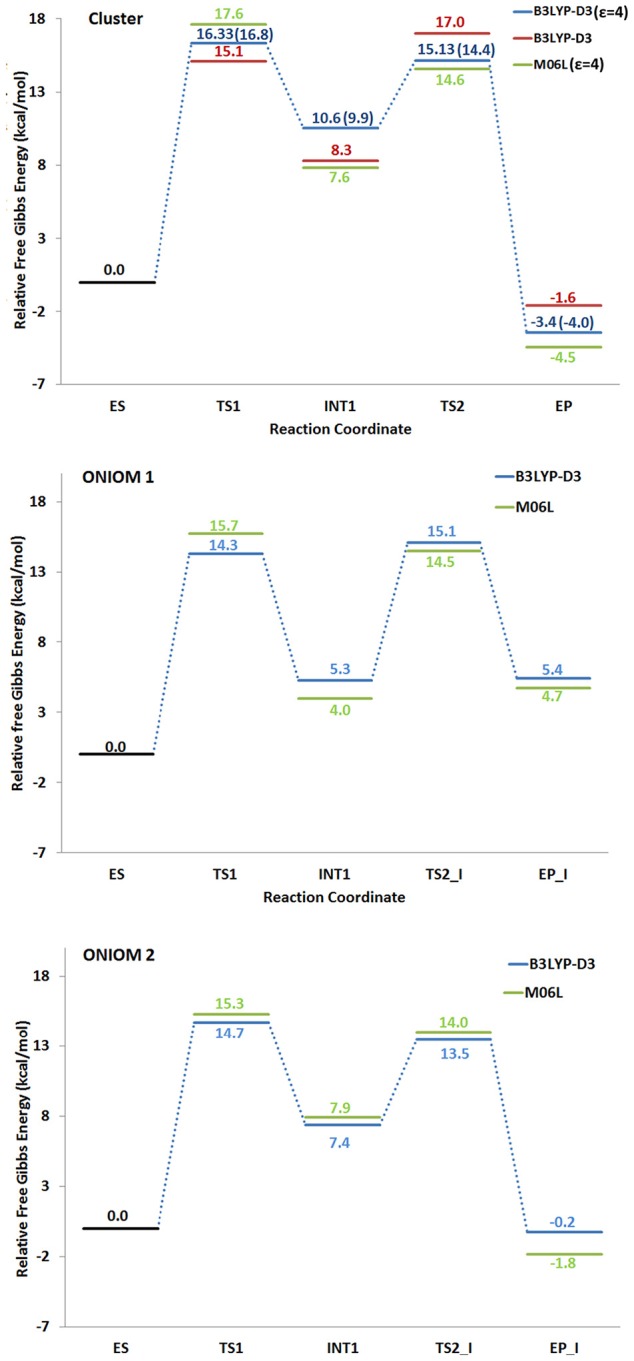
Potential energy surfaces obtained with different density functionals. The numbers in paranthesis correspond to those obtained in a previous larger QM cluster study (Sheng et al., 2017).

**Figure 4:**
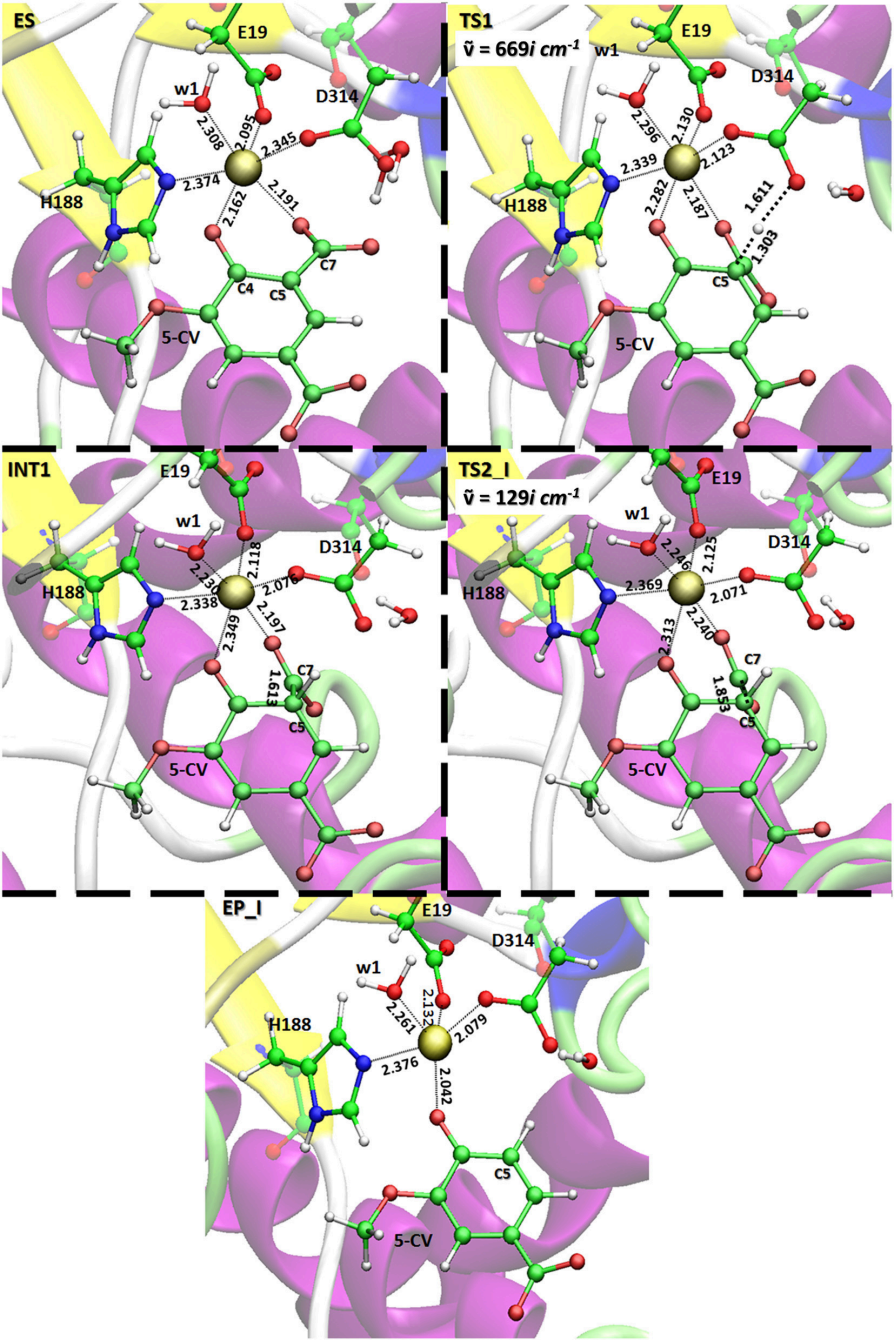
Optimized QM layer in ball and sticks (ONIOM 2 model) of all species present on the potential energy surface obtained with the B3LYP/6-31G(d,p)|SDD:FF99SB level. For clarity, only the amino acid residues of the inner coordination shell of the metal center are retained. The distances are indicated in Å. Imaginary frequencies of the transition states are also reported.

All the obtained PESs with the used models are depicted in Figure 3. Those concerning the QM one will be compared with the values arising from the previous larger QM-cluster model study (Sheng et al., 2017). B3LYP optimized structures obtained employing the ONIOM-2 model of all the species are given in Figure 4 while that for the QM and ONIOM-1 models are given in Figures S2, S3.

In **ES** complex, the Asp314, as in the original X-ray structure, is oriented in a suitable way to deliver the proton to C5 of the substrate (H_Asp314_-C5 3.103 Å). **w2** and **w3** water molecules originally bonded to the metal ion and displaced upon the substrate entrance, lie in proximity to the reaction site establishing H- bonds network with the surrounding amino acid residues (see Figure S4). The bond lengths in the active site of the present (126 atoms) and previous (Sheng et al., 2017) larger (308 atoms) QM cluster study agree very well.

The formation of **INT1** takes place through the transition state **TS1** that describes the proton transfer from the Asp314 to the carbon atom of 5-CV. The related imaginary frequency (669*i* cm^−1^) well accounts for this process since it is associated to the stretching vibrational motions of the proton transfer (O–H and H–C5). The analysis of the **TS1** optimized structure (Figure 4 for ONIOM-2 and Figure S2 for QM cluster) reveals that the formation of the C5-H bond (1.303 Å) is more advanced in the case of ONIOM-2 calculation. In fact, the breaking bond between hydrogen and oxygen of Asp314 (1.611 Å) is more elongated than the usual sp^3^ O-H bond. Furthermore, a major distortion of the −COO^−^ moiety out of plane of the phenyl ring of the substrate can be observed (76 degrees in ONIOM-2 vs. 19 degrees in QM cluster). These geometrical differences may be responsible from the slight variations in the **TS1** barrier (14.7 kcal/mol and 16.3 kcal/mol for ONIOM-2 and QM cluster, respectively). **INT1** (Figure 4) is characterized by a C5-C7 single bond with a distance slightly elongated (1.613 Å) with respect to the single canonical bond (C-C) and a sp^3^ C5 hybridized prone for the subsequent decarboxylation step. The barrier for the **CO**_2_ formation (**TS2_I**) is calculated to be 13.4 kcal/mol above **ES** complex, (only 6 kcal/mol relative to the **INT1**). The present QM cluster model obtains this barrier as 15.1 kcal/mol, analogous to the result (14.4 kcal/mol) of the previous (Sheng et al., 2017) cluster study with larger QM size.

The **TS2_I** is characterized by the C5-C7 distance of 1.853 Å associated with a relative imaginary C-C stretching frequency of 129*i* cm^−1^ (Figure 4). The already formed carbon dioxide is still coordinated to the metal ion (2.240 Å) and the manganese ion is still hexa-coordinate in octahedral geometry fashion (Figure 4). This topology is present in all our used models and in the previous larger QM cluster. (Sheng et al., 2017) At the end of the decarboxylation process, one molecule of carbon dioxide is released and the **EP_I** complex is generated (see Figure 4). The manganese ion assumes a trigonal bipyramidal geometry due to the loss of the sixth ligand (**CO**_2_). The created vacancy will be filled by one of the two water molecules present in active site (**w2** and **w3**) and essential to restore the catalytic cycle. ONIOM-2 offers a better value of the reaction energy (0.2 kcal/mol below the **ES** complex, see Figure 3) while at QM level it is exergonic (−3.5 kcal/mol, see Figure 3). In order to verify the role of the bulk potential on the cluster model, single point computations were performed on the previous optimized structures removing all the environmental effects. Results, reported in Figure 3, show that the PES behavior is almost retained. The largest effect (−2.3 kcal/mol) concerns the INT1 species.

**Figure 5:**
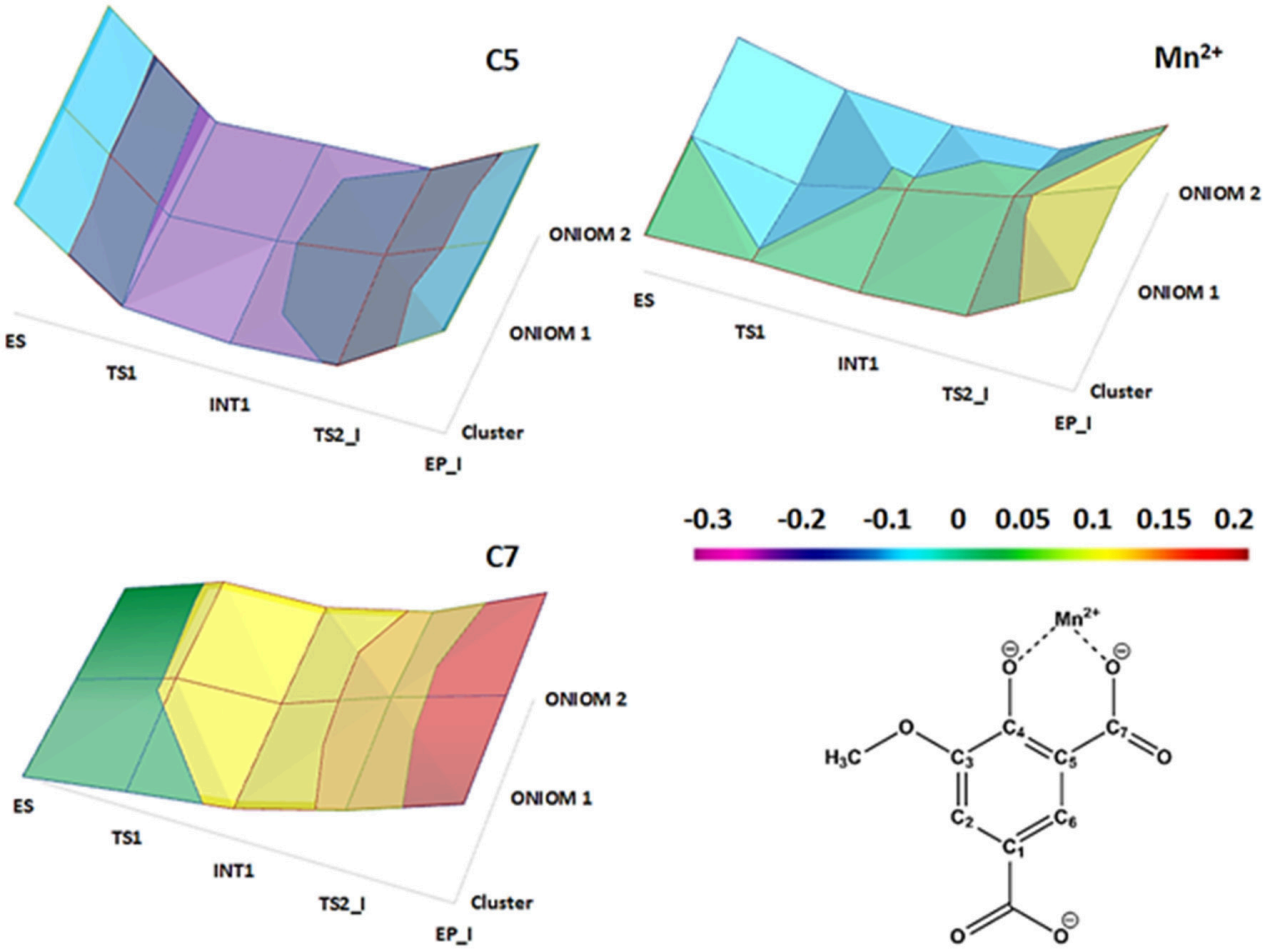
NBO charge distributions on the C5, C7, and Mn^2+^ centers directly involved in the reaction steps for the three models used.

NBO charges trend illustrated in Figure 5 confirms the nonoxidative nature of the decarboxylation process as evinced from the average value of the charges of the Mn^2+^ (1.117 *e*), the C5 (−0.417 *e*) and the C7 (0.918 *e*) atoms in all the species intercepted on the PES. From the Figure S5, it can be also evidenced that the nonbonded interactions (characterizing the amino acid residues of the inner coordination shell with the metal ion) as well as the stacking interactions between the substrate (product) and Tyr317 are retained during the reaction.

All the models propose the **TS1** which describes the formation of the C5-protonated intermediate, as the rate limiting step (14.7 kcal/mol). Based on the experimental k_cat_ value of 27 s^−1^ for *Sphingomonas paucimobilis* LigW (Sheng et al., 2017), the reaction barrier is expected to be ~16 kcal/mol. The closeness of the experimental estimate of the reaction barrier and computational **TS1** barrier suggest the appropriateness of the present and previous computational protocols.

The optimized species intercepted along the PES for the bicarbonate release (step II) are shown in Figure S6. The **w3** molecule comes into play in the reaction since it performs a nucleophilic attack on the carbon (C7) (O_w3_ – C7 distance of 1.944 Å) for generating the ${\text{HCO}}_{3}^{-}$ species and simultaneously donating a proton to Asp314 (H_w_ – O_Asp314_ distance of 1.532 Å). The obtained energy barrier is 30.3 kcal/mol (see Table S1).

## Conclusion

In this work, we have investigated the reaction mechanism of LigW by using three different models and two exchange correlation functionals. This allowed us to assess the influence of the employed model on the computed structures and energetics compared with available experimental data. The models include full structure, its partial solvation, and reactive center with the rest represented by a bulk potential including geometrical restraints at the border. Since the results of these three models, previous larger QM cluster and experimental studies are consistent to each other, the amino acids and waters outside the reactive center act on the reaction energetics in an average way for the present enzyme system. A similar behavior was also observed in many other enzymes (Himo, 2006, 2017; Blomberg et al., 2014). However, one should keep in mind that every enzyme system acts differently and thus one should avoid the generalization of the result despite its validity on a large variety of enzyme systems.

## Author contributions

MP, TM, and NR have analyzed the results, edit and reviewed equally the manuscript. MP, TM, and NR approved it for publications.

### Conflict of interest statement

The authors declare that the research was conducted in the absence of any commercial or financial relationships that could be construed as a potential conflict of interest.

## Footnotes

^1^Gaussian 09, Revision D.01 (2011), *Gaussian*, Inc., Wallingford CT.

^2^NBO, version 3.1 (2001).

^3^NCIPLOT, version3.0 (2011). Download: http://www.lct.jussieu.fr/pagesperso/contrera/nciplot.html

^4^AMBER 16 (2016), University of California, San Francisco.
