# Supplementary material for: QM Cluster or QM/MM in Computational Enzymology: The Test Case of LigW-Decarboxylase
Source: Front Chem. 2018 Jun 28;6:249. doi: 10.3389/fchem.2018.00249 (PMC6031855; doi:10.3389/fchem.2018.00249)
Supplement: Supplementary file 1 [file Presentation_1.PDF]

Supplementary Material

QM cluster or QM/MM in computational enzymology: the test case of LigW-decarboxylase

Mario Prejanò[1],Tiziana Marino[1*], Nino Russo[1]

*** Correspondence:** Tiziana Marino: tiziana.marino65@unical.it

Supplementary Data

Tab. S1 Energy values of all the species present on the potential energy surfaces of two explored paths.

	Cluster		ONIOM 1		ONIOM 2	
	SMD(ε=4)6-311+G(2d,2p)|SDD		6-311+G(2d,2p)|SDD: amber		6-311+G(2d,2p)|SDD:amber	
	B3LYP-D3	M06-L	B3LYP-D3	M06-L	B3LYP-D3	M06-L
**ES**	0.0	0.0	0.0	0.0	0.0	0.0
**TS1**	16.3	17.6	14.3	15.7	14.7	15.3
**INT1**	10.6	7.9	5.3	4.0	7.4	7.9
**TS2_I**	15.1	14.6	15.1	14.5	13.5	14.0
**EP_I**	-3.4	-4.5	5.4	4.7	-0.2	-1.8
**TS2_II**	34.2	41.6	43.9	46.3	30.3	31.9
**EP_II**	3.9	-7.9	12.6	11.7	-6.9	-4.8

Tab. S2. Calculated parameters for 5-carboxyvanillate.

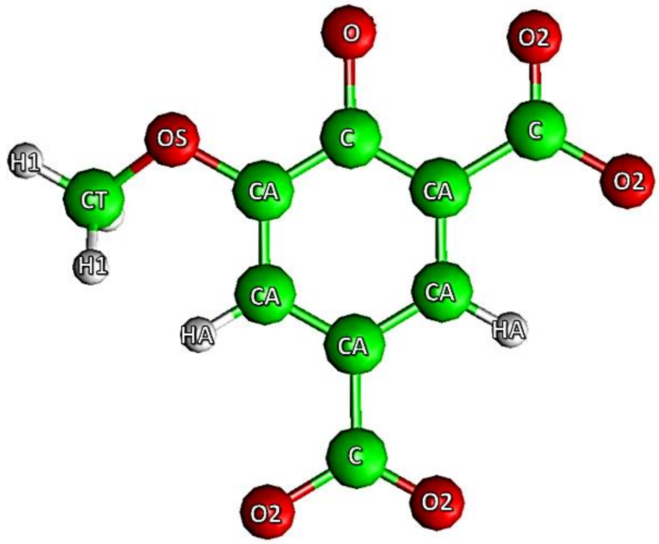

Atomtype	Charge
CA	-0.266
OS	-0.443
CT	0.135
H1	0.007
HA	0.120
C	0.974
O2	-0.896
O	-0.786

Bond	K_i / kcal mol^{-1} Å^{-2}	l_0/Å
CA-CA	478.40	1.387
CA-C	449.90	1.406
CA-HA	344.30	1.087
CA-OS	392.60	1.357
OS-CT	301.50	1.439
CT-H1	337.30	1.092
C-O2	648.00	1.214
C-O	648.00	1.214

Angle	K_{an}/ Kcal mol^{-1} rad^{-2}	θ_0/deg
CA-CA-CA	67.180	119.970
CA-CA-HA	50.300	119.700
CA-C-O2	72.770	119.120
CA-CA-C	67.930	120.700
CA-CA-OS	71.040	121.890
CA-OS-CT	64.210	112.090
CA-C-CA	67.170	116.780
CA-C-O	72.770	119.120
OS-CA-C	70.229	117.340
OS-CT-H1	50.870	108.700
H1-CT-H1	39.430	108.350
O2-C-O2	78.170	130.380
C-CA-C	66.570	118.880

Dihedral	d	V_n/	γ/deg	n

		Kcal mol^{-1} rad^{-2}		
CA-CA-CA-OS	1	6.650	180.000	2.000
CA-CA-CA-C	1	6.650	180.000	2.000
CA-CA-C-O2	1	2.175	180.000	-2.000
CA-CA-C-O2	1	0.300	0.000	3.000
CA-CA-CA-HA	1	6.650	180.000	2.000
CA-CA-CA-CA	1	3.625	180.000	2.000
CA-CA-OS-CT	1	1.050	180.000	2.000
CA-CA-C-CA	1	2.175	180.000	2.000
CA-CA-C-O	1	2.175	180.000	-2.000
CA-CA-C-O	1	0.300	0.000	3.000
CA-OS-CT-H1	1	0.383	0.000	3.000
CA-C-CA-C	1	2.175	180.000	2.000
OS-CA-CA-HA	1	6.650	180.000	2.000
OS-CA-C-CA	1	2.175	180.000	2.000
OS-CA-C-O	1	2.175	180.000	2.000
CT-OS-CA-C	1	1.050	180.000	2.000
HA-CA-CA-C	1	6.650	180.000	2.000
C-CA-C-O	1	2.175	180.000	2.000
O2-C-CA-C	1	2.175	180.000	2.000

Improper	V_n/ Kcal mol^{-1} rad^{-2}	γ/deg	n
C-CA-CA-CA	1.1	180.0	2.0
CA-CA-CA-HA	1.1	180.0	2.0
C-CA-CA-OS	1.1	180.0	2.0
CA-O2-C-O2	1.1	180.0	2.0
C-C-CA-CA	1.1	180.0	2.0
CA-CA-C-O	1.1	180.0	2.0

VdW	r_0 /Å	ε/ kcal mol^{-1}
CA	1.9080	0.0860
OS	1.6837	0.1700
CT	1.9080	0.1094
H1	1.4870	0.0157
HA	1.4870	0.0157
C	1.9080	0.0860
O2	1.6612	0.2100
O	1.6612	0.2100

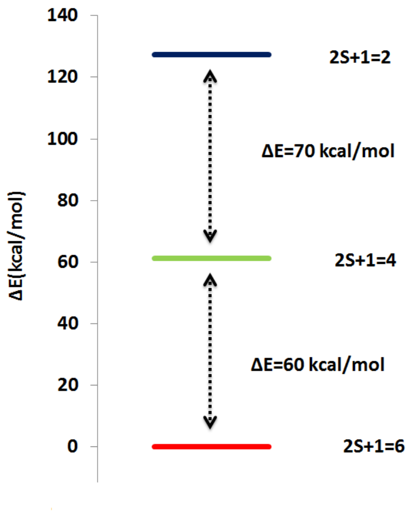

Fig. S1 Energetic behavior of the ES species at the considered spin multiplicities.

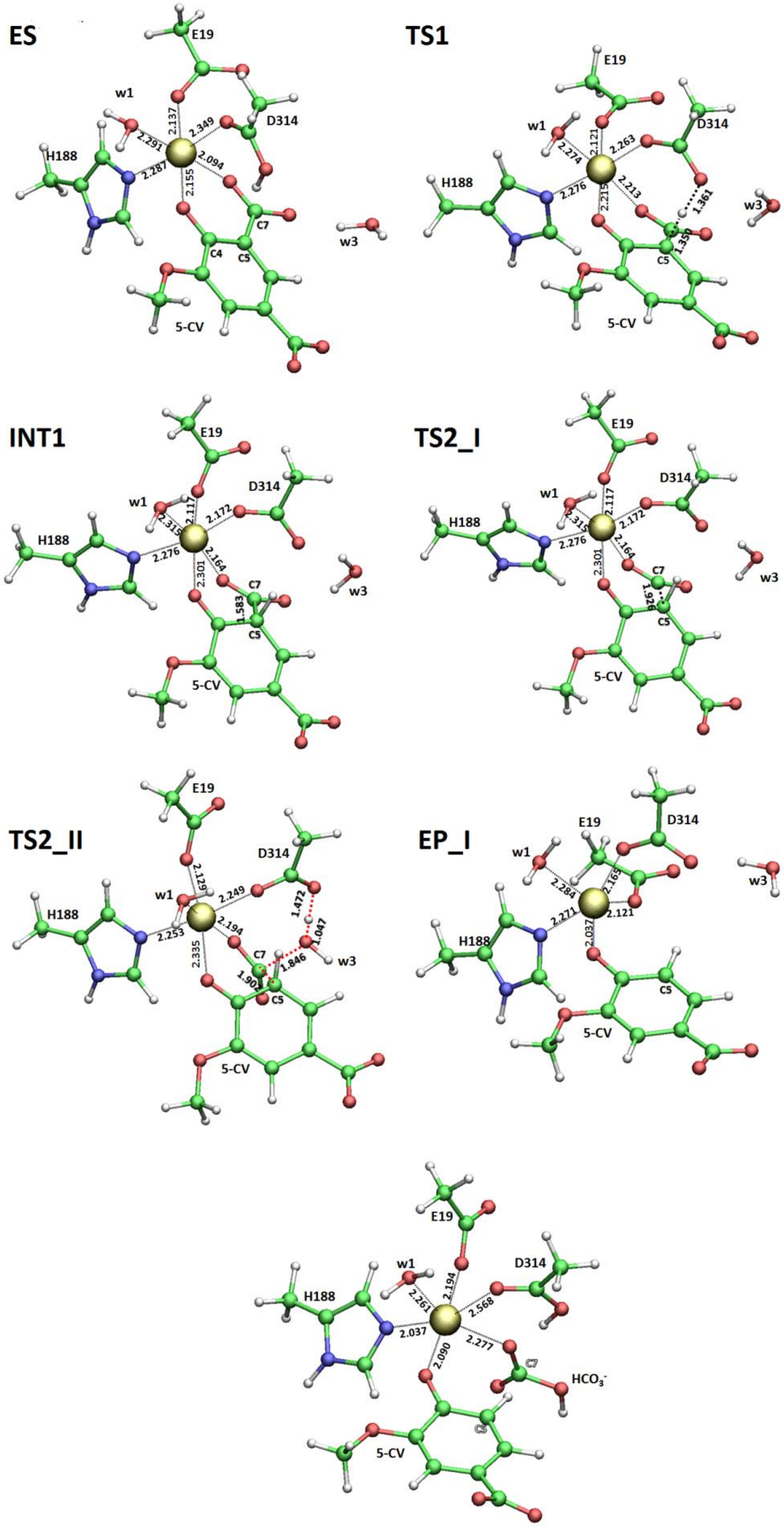

Fig. S2 B3LYP/6-31+G(d,p) (SDD for Mn) optimized geometries in QM-cluster for both explored paths.

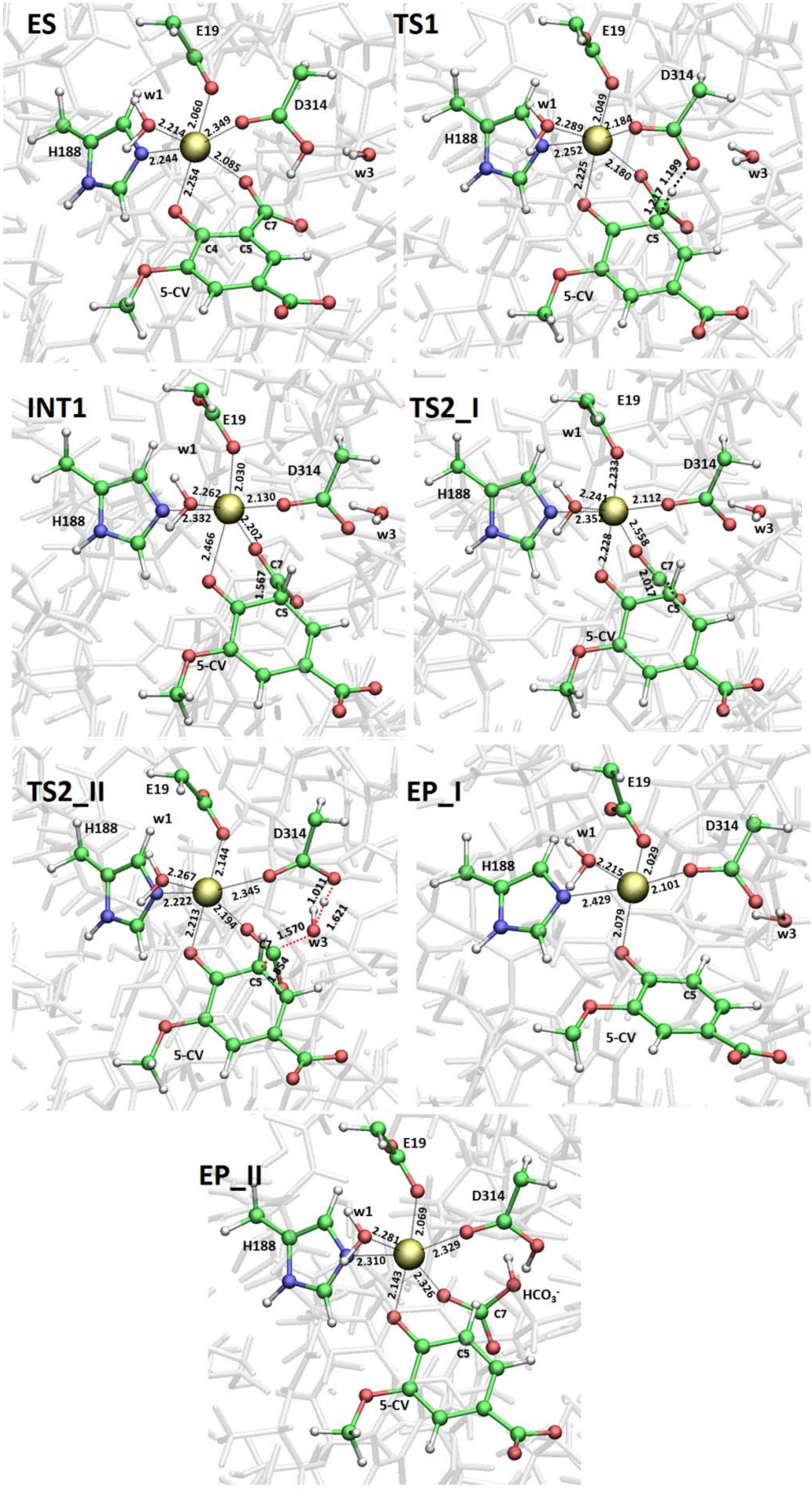

Fig. S3 ONIOM 1 optimized geometries for both explored paths.

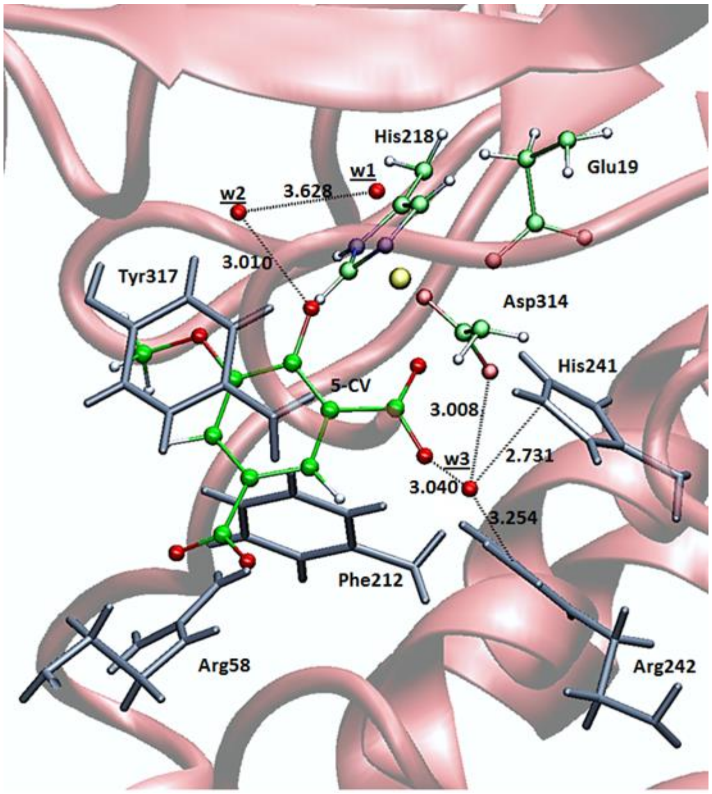

Fig. S4 Water molecules (**w2** and **w3**) implicated in the hydrogen bonds network in ES complex (ONIOM-2).

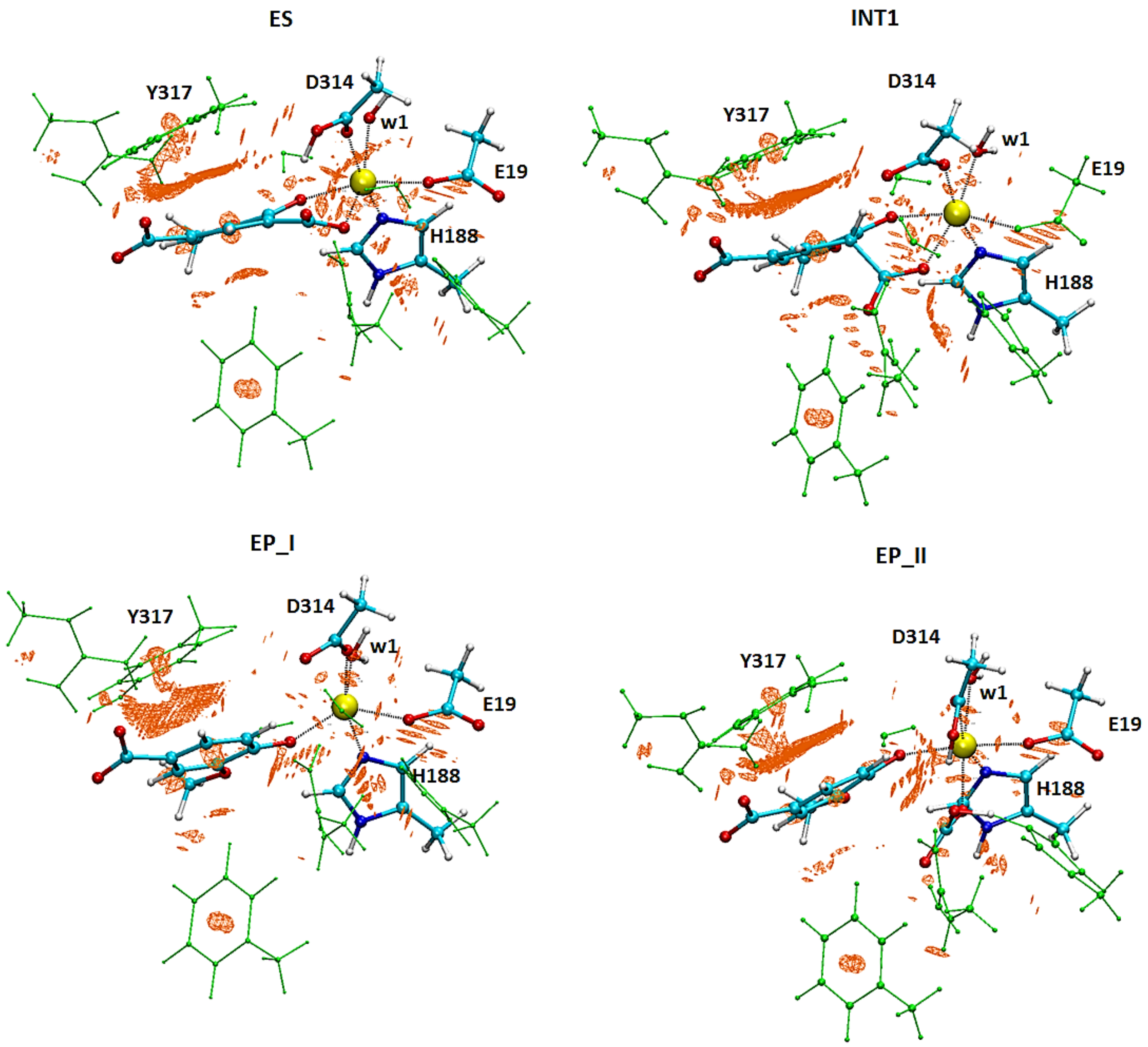

Figure S5. Nonbonding interactions plot (orange isosurfaces) calculated for the minima intercepted on the PES.

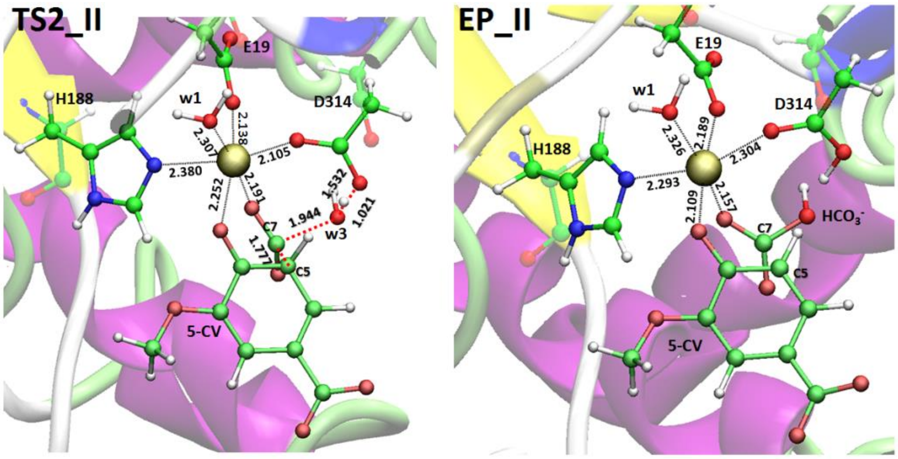

Fig. S6. ONIOM 2 optimized geometries of the characterized species present on the HCO_3^- formation path.
